# A challenging discrimination of an intensely [^18^F]PSMA-1007-avid solitary lesion at the skull base in a patient with biochemical recurrence of prostate cancer

**DOI:** 10.1093/bjrcr/uaae041

**Published:** 2024-11-01

**Authors:** Emil Novruzov, Günter Niegisch, David Pauck, Dominik Schmitt, Julian Kuhlmann, Kerim Beseoglu, Gerald Antoch, Lars Schimmöller, Frederik L Giesel, Eduards Mamlins

**Affiliations:** Department of Nuclear Medicine, Medical Faculty and University Hospital Duesseldorf, Heinrich-Heine-University Duesseldorf, 40225 Düsseldorf, Germany; Department of Urology, Medical Faculty and University Hospital Duesseldorf, Heinrich-Heine-University Duesseldorf, 40225 Düsseldorf, Germany; Institute of Neuropathology, Medical Faculty and University Hospital Duesseldorf, Heinrich-Heine-University Duesseldorf, 40225 Düsseldorf, Germany; Department of Nuclear Medicine, Medical Faculty and University Hospital Duesseldorf, Heinrich-Heine-University Duesseldorf, 40225 Düsseldorf, Germany; Department of Nuclear Medicine, Medical Faculty and University Hospital Duesseldorf, Heinrich-Heine-University Duesseldorf, 40225 Düsseldorf, Germany; Department of Neurosurgery, Medical Faculty and University Hospital Duesseldorf, Heinrich-Heine-University Duesseldorf, 40225 Düsseldorf, Germany; Department of Diagnostic and Interventional Radiology, Medical Faculty and University Hospital Duesseldorf, Heinrich-Heine-University Duesseldorf, 40225 Düsseldorf, Germany; Department of Diagnostic and Interventional Radiology, Medical Faculty and University Hospital Duesseldorf, Heinrich-Heine-University Duesseldorf, 40225 Düsseldorf, Germany; Department of Diagnostic, Interventional Radiology and Nuclear Medicine, Marien Hospital Herne, University Hospital of the Ruhr-University Bochum, 46625 Herne, Germany; Department of Nuclear Medicine, Medical Faculty and University Hospital Duesseldorf, Heinrich-Heine-University Duesseldorf, 40225 Düsseldorf, Germany; Department of Nuclear Medicine, Medical Faculty and University Hospital Duesseldorf, Heinrich-Heine-University Duesseldorf, 40225 Düsseldorf, Germany

**Keywords:** PSMA imaging, PSMA-1007, [^18^F]PSMA-1007, meningioma, tumour-to-meningioma, prostate cancer, brain metastasis, skull base

## Abstract

Prostate adenocarcinoma metastasis to brain has been reported to occur only up to 0.6% of patients and these are mostly diagnosed in autopsy series. In the setting of biochemical recurrence of prostate cancer, a suspected PSMA-avid (prostate-specific membrane antigen) lesion in the brain is still strongly suggestive of an intracranial metastasis of prostate cancer. This needs, however, a thoroughly recurrency work-up due to other potentially PSMA-avid cranial lesions, as PSMA initially was developed for the imaging of primary CNS tumours. We report of a challenging clinical case of a 71-year-old-patient with a strongly PSMA-avid lesion at the skull base. Given the medical history of a meningioma at the skull base, the further diagnostic work-up with MRI could still not rule out a malignancy, so that the patient needed to undergo a surgical excision of the tumour mass. The histological and immunohistochemical examinations revealed a relapsed CNS WHO grade 1 meningioma. From the aspect of molecular imaging and critical analysis of regular clinical care in a third-level university hospital, we consider this result very intriguing. Hence, we analyse the decision-making process and clinical course of this case in the light of molecular imaging findings.

## Introduction

**Figure 1.**
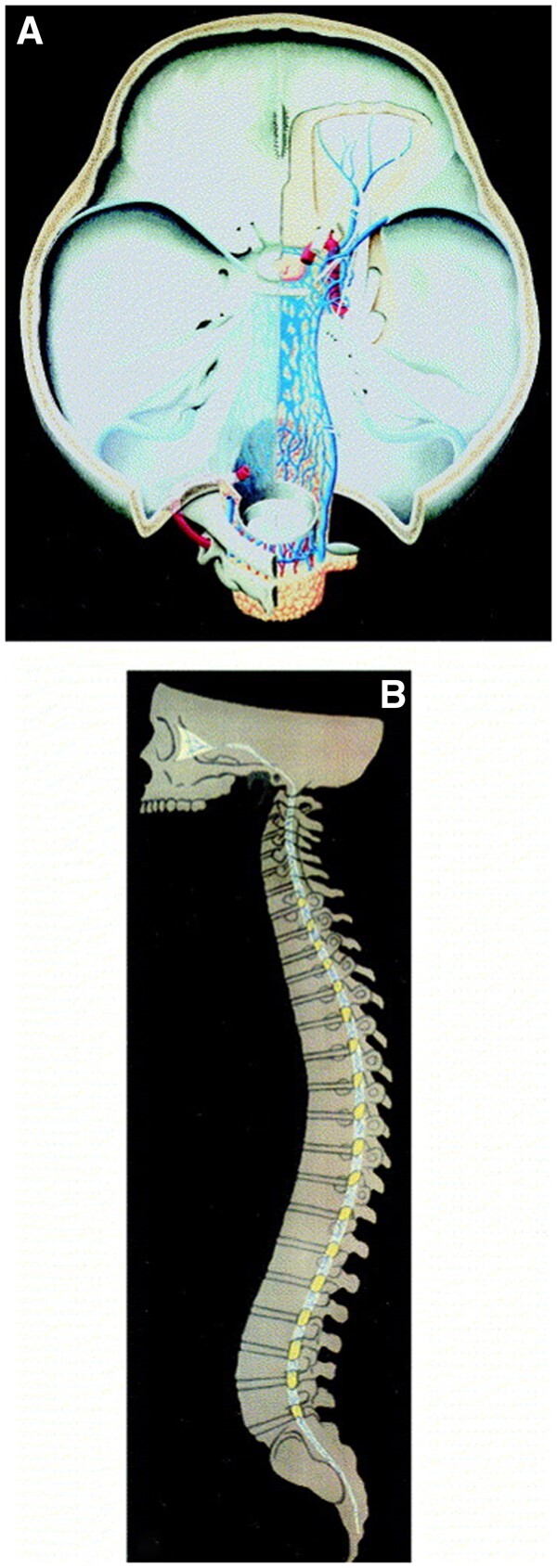
(A) Schematic drawing to illustrate the continuation of the venous and adipose structures from the orbit to the lateral sellar compartment on down the clivus and basi-occiput (Breschet’s veins) to Batson’s veins. (B) Depiction of the midline sagittal section of the entire neural axis, indicating veins and fat from orbit to coccyx continuously within the extradural compartment (adapted from Parkinson^3^).

Prostate cancer represents the second most common malignancy in men with a still substantial morbidity worldwide due to high recurrence rates of up to 53% after the initial curative-intent therapy. Following the introduction of PSMA PET ligands, PSMA PET imaging has evolved to the backbone of the recurrence work-up in the context of biochemical recurrence (BCR) of prostate cancer with elevated tumour marker levels (prostate-specific antigen, PSA). The usual sites of recurrence tend to be local relapse in the prostate bed or osseous and lymph node metastases in the pelvis.^1^ In early cancer stages intracranial metastases have been reported to occur very seldom. Only up to 0.6% of all metastases of prostate cancer and, according to autopsy cases, 3% of brain parenchyma metastases were reported to occur from prostate cancer.^2^ The spread of cranial or leptomeningeal metastases of prostate cancer has been demonstrated to occur through the connection of prostatic venous plexus and cerebrospinal venous system (CSVS) within the extradural neuraxis compartment which is described as an intricate, extradural, neuraxial continuum consisting of adipovenous tissue, epidural veins, and traversing neurovascular structures between the orbit and the coccyx (Figure 1). Hence, prostate cancer metastasis may involve all skull base fossae via this anatomic continuum provided by the CSVS and mimic a variety of central nervous system (CNS) tumours at the skull base.^3^^,^^4^

We aimed to describe the clinical course of a case with a highly suspected metastasis of prostate cancer at the skull base in a 71-year-old-patient and to discuss the background of challenging molecular imaging features.

## Clinical presentation

**Figure 2.**
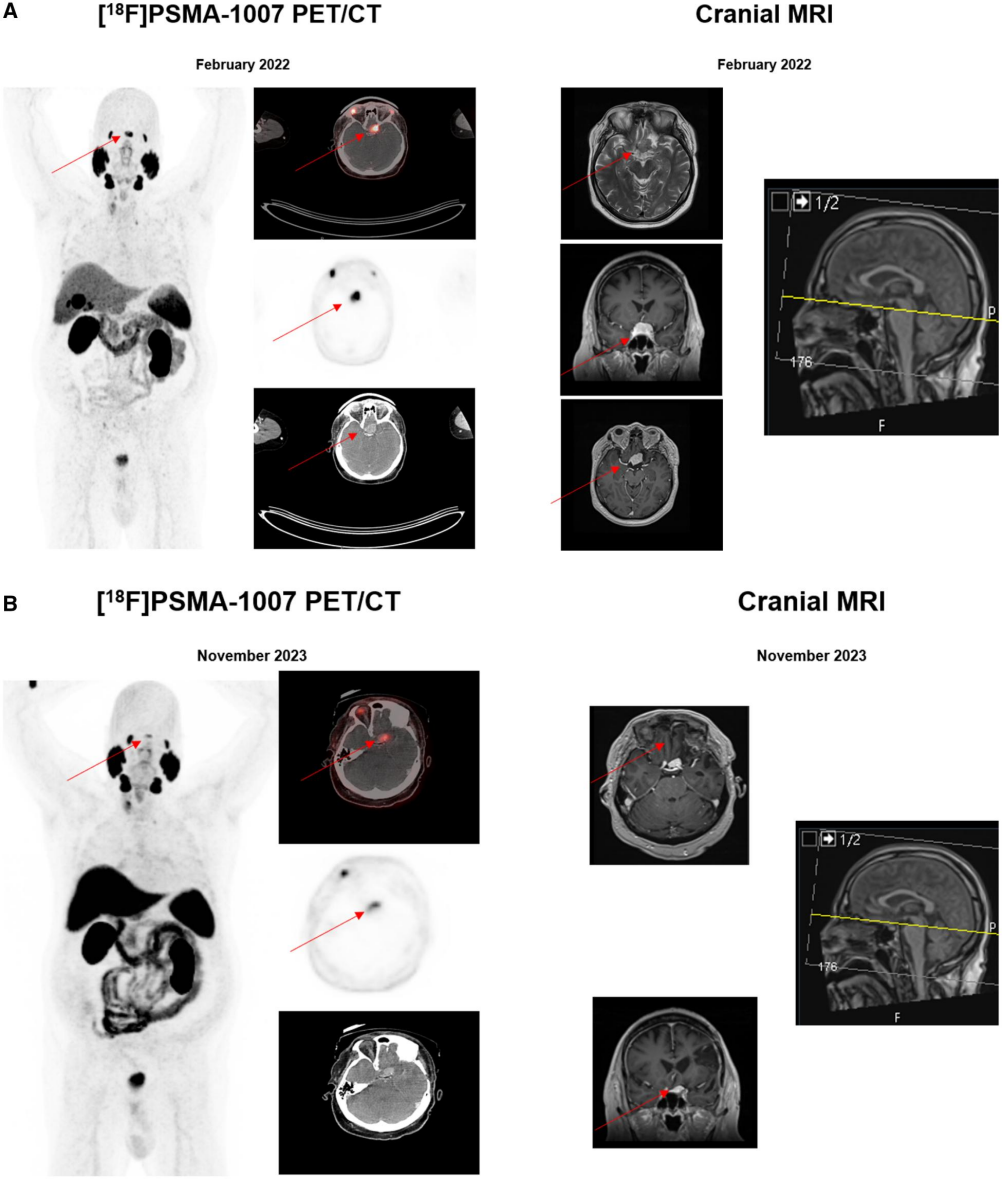
(A) The pre-operative imaging with [^18^F]PSMA-1007 PET/CT and cranial MRI findings within recurrency work-up revealed a solitary, intensely PSMA-avid (SUV_max_ value of 24.9), 2.6 × 1.9 × 1.3 cm mass at the skull base with compression of the right optic nerve. (B) Despite a continuously increasing PSA level up to 4.0 ng/mL, further follow-up examinations with [^18^F]PSMA-1007 PET/CT and cMRI scans could not reveal any sign for a malignant focus. The only remarkable finding was a moderately PSMA-avid, suprasellar residual meningioma with SUVmax value of 8.4.

**Figure 3.**
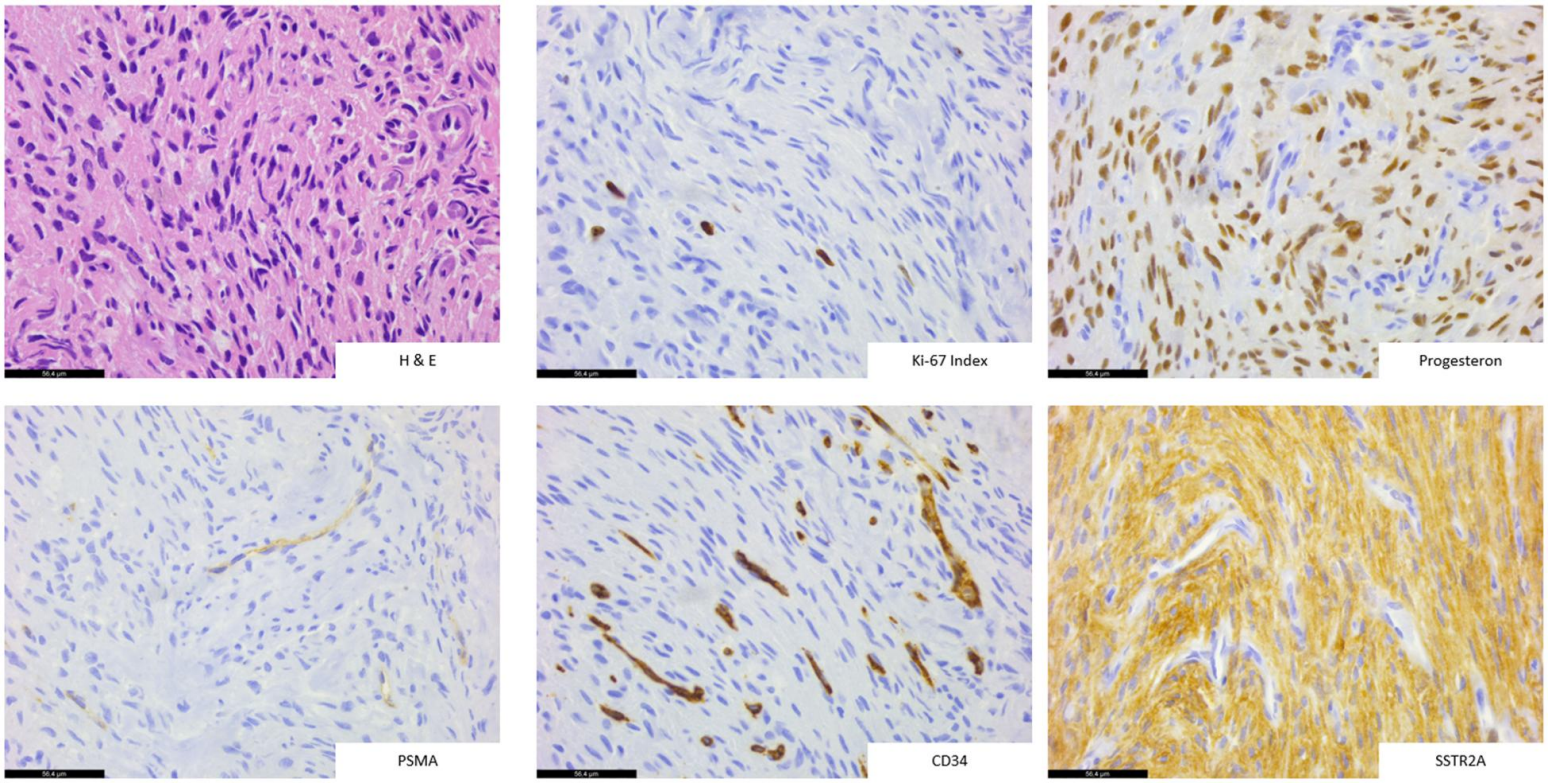
The histological and immunohistochemical examination of the neurosurgically resected intracranial tumour mass exhibited intense expression of SSTR2A and moderate expression of progesterone receptors, while PSMA expression was found to be markedly low. Additionally, the Ki-67 (MIB1) index was lower than 5%, consistent with a recurrent CNS WHO grade 1 meningioma.

The 71-year-old patient presented in February 2022 due to BCR with an elevated PSA level of 0.6 ng/mL after a radical prostatectomy operation for prostate cancer with a Gleason score of 7 b (ISUP 3) in June 2014. In the past, the patient had undergone the resection of meningioma with decompression of the left optic nerve at the skull base resulting in anopsia of the left eye as a postoperative complication in October 2013. The whole-body, contrast-enhanced [^18^F]PSMA-1007 PET/CT scan performed in February 2022 revealed no evidence for a local relapse or any osseous or lymph node metastases. However, our examination exhibited a solitary, intensely PSMA-avid, 2.6 × 1.9 × 1.3-cm mass at the skull base with compression of the right optic nerve. The SUV_max_ value was at 24.9 and, therefore, the lesion was considered highly suspicious for a solitary cranial metastasis of prostate cancer. Given the past history of meningioma and previous follow-up cranial magnetic resonance imaging (cMRI) results with residual meningioma lesion at the skull base, we performed further diagnostic work-up with a cMRI a couple of weeks later that correspondingly showed a T2-hyperintense, homogeneously contrast-enhancing, mildly lobulated, progressive tumour mass, lying left paramedial to the sella turcica and adjacent to the planum sphenoidale with contact to the optic chiasm (Figure 2A). The interpretation of cMRI signs could not exclude malignancy. The following discussions in our in-house multidisciplinary uro-oncological and neuro-oncological tumourboards, in February and May 2022 respectively, recommended the decision of neurosurgical resection of the tumour mass. Thus, the patient underwent a neurosurgical partial resection of the tumour mass with decompression of right optic nerve in July 2022. The histological examination demonstrated meningotheliomatous differentiated tumour cells with round to oval, medium-sized nuclei and a loosened chromatin structure as well as pale eosinophilic, poorly demarcated cytoplasm. Besides moderate to high expression of progesterone and SSTR2A receptors, PSMA staining was restricted to low expression in the endothelial cells of tumour vessels and the Ki-67 level was <5%. The histological examination of the resected mass was consistent with a relapse of CNS WHO grade 1 meningioma (Figure 3). The patient showed a reasonable postoperative recovery despite a transient aphasia and permanent hemianopsia of right eye. The patient received stereotactic radiotherapy with a total radiation of 54 Gy in 18 fractions given the gradual postoperative growth of the residual tumour with proximity to left ACI in January 2023. Despite a continuously increasing PSA level up to 4.0 ng/mL, the recurrence work-up with repeated whole-body contrast-enhanced [^18^F]PSMA-1007 PET/CT and cMRI scans at further follow-up in November 2023 did not show a malignant focus, but a moderately PSMA-avid, suprasellar residual meningioma with an SUV_max_ value of 8.4. Consequently, the situation appeared to be consistent with the so-called PSMA negative BCR of prostate cancer, thus an androgen-deprivation therapy is initiated in November 2023 (Figure 2B).

### Likely differential diagnoses on the basis of recurrence work-up

- Solitary CNS metastasis of prostate cancer
- Tumour-to-tumour metastasis (TTM) of prostate cancer
- A local relapse of meningioma

The correct diagnosis could only be made based on histopathological validation after neurosurgical subtotal resection of tumour mass at the skull base.

## Discussion

We present a case of local relapse of CNS WHO grade I meningioma at the skull base with molecular and radiological imaging features suggestive of a distant metastasis of prostate adenocarcinoma, as the tumour mass exhibited an intense [^18^F]PSMA-1007 uptake and features of malignancy in cMRI. Meningiomas are mostly benign and originate from arachnoid cap cells of meningeal membrane accounting for around 36% of all CNS tumours. The majority of meningiomas are benign (CNS WHO grade 1), while approximately 10%-15% have a more aggressive course and tend to display rapid tumour growth, recurrence or metastatic disease (CNS WHO grades 2 and 3). It is evident that PSMA expression of several tumours demonstrates a positive correlation with tumour-driven neo-angiogenesis. PSMA is a 100-kDa transmembrane peptidase upregulated primarily in prostate cancer cells as well as in endothelial cells of solid tumours of the breast, lung, thyroid, pancreas, and urothelium, including sarcomas and primary glial tumours. This is probably due to the enzymatic activity, whereas the angiogenesis process in normal tissue does not seem to involve PSMA expression. Therefore, any lesion with a relevantly increased, incidental PSMA uptake in molecular imaging potentially indicates a malignant angiogenesis and requires further diagnostic work-up in regular clinical care to rule out malignancy. Given the high vascularization of meningiomas, the degree of PSMA expression within this entity has been elusive. To date, there are few reports of slight to moderate PSMA uptake of meningiomas with a SUV_max_ value of up to 12.0 that mostly lacked a histopathological validation.^5^^,^^6^

Tubre et al systematically investigated, to the first time, the characteristics and correlation of PSMA uptake in the meningioma to elucidate the potential of PSMA directed diagnostics and therapy for this entity on the basis of serial histopathological examinations. They reported basically all meningioma specimens to express PSMA within their endothelial cells and this would positively correlate within recurrent tumours or tumours with a prior radiotherapy, albeit no correlation was observed between the degree of PSMA expression and specific de novo meningioma grades.^6^

In the light of the few previous case reports and especially the work of Tubre et al, our case underscores important insights in PSMA imaging of recurrent meningiomas. The immunohistochemical examination exhibited in total a low overall PSMA expression despite a highly intense [^18^F]PSMA-1007 uptake with a SUV_max_ value of 24.9 (Figure 3). This indicates the intact PSMA expression in tumour vasculature through the neo-angiogenesis, although the recurrency of tumour did not appear to have the raw PSMA expression upregulated as the results of Tubre et al predicted. The previous case reports with incidental PSMA uptake of meningiomas have mostly deployed ^68^Ga-PSMA-11 with an SUV_max_ interval between 1.9 and 14.6.^5^ Among previous reports, Haemels et al were the only research group to report cranial meningioma with incidental [^18^F]PSMA-1007 uptake and histological validation, which exhibited only a low-to-moderate PSMA uptake on PET/CT scan as well.^7^

In view of these facts and the results of recurrency work-up, a clinical interpretation of the imaging findings as a malignancy was inevitable, as this might suggest either the so-called TTM or distinct metastatic spread to the proximity of the location of a former meningioma at the skull base. TTM is a rare phenomenon which describes metastatic spread to benign tissue, for example, meningioma, with distinct hallmarks such as lipid- and collagen rich content with slow-growth pattern embedded within abundant vascularity. This combination of features would provide a fertile soil for the seeding of malignant cells.^8^^,^^9^ We considered the direct metastatic spread in the vicinity of the location of former meningioma as an alternative differential diagnosis, while a relapsed meningioma was considered to be unlikely. Particularly the highly intense [^18^F]PSMA-1007 uptake of lesion reinforced our hypothesis of malignancy. Therefore, as Rzehak et al pointed out, surgery is the only valid method to ensure an accurate diagnosis for vague cases of TMM, as clinical and radiological findings are neither specific nor sensitive enough for this purpose. Remarkably, the follow-up examination with cMRI and [^18^F]PSMA-1007 PET/CT two years later demonstrated still viable, small-size residual spheno-orbital meningioma with only a moderate PSMA uptake of SUV_max_ 8.0 (Figure 2).

Given the lower overall PSMA expression in immunohistochemistry, the only reasonable explanation for the contradictory PSMA and histological findings of our case seem to lie in the fact that high PSMA uptake on PET/CT does not necessarily indicate high PSMA expression in the tumour tissue, but it is rather influenced by other factors such as vascular density, blood flow, or nonspecific ligand binding. In conclusion, the interpretation of PSMA imaging requires a comprehensive analysis and correlation of the patient's medical history, clinical findings, and patterns as well as pitfalls of molecular imaging to enable accurate clinical decision-making.

## Learning points

- Tumour-to-meningioma or direct intracranial metastatic spread of prostatic adenocarcinoma are very rare clinical scenarios and their accurate discrimination from benign meningiomas represents a diagnostic dilemma.
- A high semiquantitative, PSMA tracer uptake on PET/CT scan might not only suggest an upregulation of PSMA expression, but also an intensified vascular density, blood flow, or nonspecific ligand binding in the lesion of interest.
- Intracranial lesions with intense PSMA uptake require a multidisciplinary, tailored approach to provide the optimal trade-off among various therapeutic options. In suspicious cases, surgical option should be considered as the method-of-choice to ensure the accurate diagnosis.

## Data Availability

The data used and/or analysed during the current study are available from the corresponding author upon reasonable request.
